# 

**Published:** 1903-01-31

**Authors:** 


### NOTICE TO CORRESPONDENTS.

All MSS., Letters, Books for Review, and other matters intended for the Editor should be addressed THE EDITOR, "THE HOSPITAL," THE HOSPITAL BUILDING, 28 & 29 SOUTHAMPTON STREET, STRAND, W.C.

The Editor cannot undertake to return rejected MS., even when accompanied by stamped directed envelope.

**Notice to Advertisers and Subscribers.**

All Advertisements, Orders for Copies of THE HOSPITAL, and Business Communications should be addressed to **THE MANAGER, (Not to the Editor)**, THE HOSPITAL, 28 & 29 Southampton Street, Strand, London, W.C.

The Cost of Subscription, post free, is as follows.—Per week, 2½d.; per quarter, 2s. 8d.; per half-year, 5s. 3d.; per annum, 10s. 6d. Foreign:—Per annum, 15s. 2d., payable, in all cases, in advance.

**NOTICE.—Vol. XXXII. of "The Hospital" (April to September 1902) in handsome cloth binding is now on sale at the office of this Journal, price, 7s. 6d. Cases for binding the Numbers contained in this Volume 1s. 6d. Index to Vol. XXXII., 2½d., post free.**

**The Monthly Part for February will be ready on Feb. 2. Price 6d., by post 8d.**

### BOOKS RECEIVED.

H. RENSHAW.

"Pharmacy, Materia Medica, and Therapeutics." By W. Whitla, M.D.

JAMES WILLING, JUN., LIMITED.

"Willing's Press Guide, 1903."

E. MARLBOROUGH AND CO.

"Practical Hints for Travellers in the near East." By E. A. Reynolds-Ball.

GEORGE NEWNES, LIMITED.

"The Story of Alchemy." By M. M. Patteson Muir.

T. B. BROWNE.

"The Advertiser's A.B.C."

### Scraps and Gleanings.

The Royal Merchant Seamen's Orphanage has received a donation of 50 guineas from the Clothworkers' Company.

Two of the masters at the King's College, Bangkok, have died of cholera there. They died on the same day, and were both members of Oxford University.

The Governesses' Benevolent Institution have received 100 guineas from the Clothworkers' Company, half of this amount being an annual subscription and the other half a special donation towards the sum of £3,000 required for the renewal of the lease of the Home at 47 Harley Street.

At the annual meeting of the Dumb Friends League the president, Mr. G. W. E. Russell, reported that the work done during the past quarter had been of a very satisfactory nature, and each of the three branches had now a membership of about one hundred. In the course of his address Mr. Russell laid particular stress on the importance of inculcating the young, by means of example on the part of their elders, with habits of kindness to all dumb creatures.

### The Student of Medicine.

### Appointments.

**H. J. F. Badcock,** M.R.C.S., L.R.C.P.Lond., appointed District Medical Officer of the Medway Union. **Joseph P. Byrne,** L.R.C.S.I., L.R.C.P.I., appointed House Surgeon to the Dewsbury and District General Infirmary. **Maud A. Chadburn,** M.D.Lond., B.S., appointed Surgeon to the Out-Patient Department, New Hospital for Women. **J. Gray Clegg,** M.D., B.S., F.R.C.S., appointed Honorary Surgeon to the Manchester Royal Eye Hospital. **W. P. Gowland,** M.R.C.S., appointed Senior House Surgeon to the Lincoln County Hospital. **John Hay,** M.D.Vict., M.R.C.S., L.R.C.P., appointed Honorary Pathologist to the David Lewis Northern Hospital, Liverpool. **J. R. Harper,** M.R.C.S., L.R.C.P.Lond., appointed Medical Officer of Health to Barnstaple Rural District Council. **James Hindshaw,** M.B., Ch.B.Vict., appointed Assistant Medical Officer to the Nottingham Parish Workhouse. **C. C. Lall,** M.B., C.M.Edin., appointed Assistant Medical Officer to the Greenwich Union Infirmary. **Norman Macnair,** B.Sc., M.D.Glasg., M.R.C.S.Eng., L.R.C.P.Lond., F.F.P.S.Glasg., appointed Extra Honorary Dispensary Physician to the Royal Hospital for Sick Children, Glasgow. **William Mair,** M.A., M.B., Ch.B., B.Sc.Edin., D.P.H.Camb., appointed Resident Medical Officer, Leith Public Health Hospital, and Assistant to the Medical Officer of Health for Leith. **Alexander Sclanders,** M.D.Edin., appointed Medical Referee under the Workmen's Compensation Act for the Sheriffdom of Inverness, Elgin, and Nairn, to act for the County of Nairn. **V. A. Settle,** L.S.A., appointed District Medical Officer of the Kington Union. **W. Webb Shackleton,** M.D.Dubl., appointed Joint Medical Officer to the Royal Masonic Institution for Boys at Bushey, Herts. **G. Francis Smith,** M.R.C.S.Eng., L.R.C.P.Lond., appointed Joint Medical Officer to the Royal Masonic Institution for Boys at Bushey, Herts. **Gnoh Lean Tuck,** M.A., M.B., B.C.Cantab., appointed House Physician to the Brompton Hospital for Consumption, S.W. **Clement White,** M.D., B.A., B.C.Cantab., appointed Assistant Honorary Surgeon of St. Bartholomew's Hospital, Rochester.

**"The Hospital" Institutional Library.**

**"Hospitals and Asylums of the World." 4 Vols., with a Portfolio of Plans, complete £12 12s.**

**"Burdett's Hospitals and Charities, 1902." 5s.**

**"Cottage Hospitals, General, Fever, and Convalescent." 10s. 6d.**

**"Hospital Expenditure: The Commissariat." 2s. 6d.**

**"A Legal Handbook for the Use of Hospital Authorities." 2s. 6d.**

**"The Cottage Hospital Case Book or Register of Patients." 10s. and 12s. 6d.**

**"The Furnishing and Appliances of a Cottage Hospital." 6d.**

**All these are published by the Scientific Press, Ltd., and may be obtained through any bookseller, or direct from the publishers, 28 and 29 Southampton Street, Strand, London, W.C.**

THE HOSPITAL FOR SICK CHILDREN,

